# Impact of trap-related non-idealities on the performance of a novel TFET-based biosensor with dual doping-less tunneling junction

**DOI:** 10.1038/s41598-023-38651-3

**Published:** 2023-07-17

**Authors:** Iman Chahardah Cherik, Saeed Mohammadi

**Affiliations:** Department of Electrical and Computer Engineering, Semnan University, Semnan, 3513119111 Iran

**Keywords:** Biomedical engineering, Electrical and electronic engineering

## Abstract

This article presents a novel dielectric-modulated biosensor based on a tunneling field-effect transistor. It comprises a dual doping-less tunneling junction that lies above an n^+^ drain region. By employing the wet-etching technique, two cavities are carved in the gate dielectric, and with the entry of various biomolecules into the cavities, the electrostatic integrity of the gate changes, accordingly. Numerical simulations, carried out by the Silvaco ATLAS device simulator, show that including trap-assisted tunneling significantly modulate the biosensor's main parameters, such as on-state current, subthreshold swing, and transconductance and their corresponding sensitivities. We also evaluate the effect of semi-filled cavities on our proposed biosensor’s performance with various configurations. The FOMs like *I*_on_/*I*_off_ = 2.04 × 10^6^, ${S}_{{I}_{ds}}$=1.48 × 10^5^, and ${S}_{SS}$=0.61 in the presence of TAT show that our proposed biosensor has a promising performance.

## Introduction

Nowadays, the need for medical diagnostic equipment capable of rapidly detecting of newly-emerging viruses has increased tremendously. Biosensors, which can detect a vast range of diseases at the early stages, are among the most popular and most interesting equipment. Biosensors are basically categorized to label detection and label free, depends on their detection mechanism. Unlike label detection biosensors, label free devices can be more accurate and prevent unwanted side effects^1^. Their ability in detecting the neutral and charged biomolecules with high sensitivity is a significant advantage compared to other biosensors, such as ion-sensitive devices^2,3^.

In^4^ authors have stated that biosensors based on tunneling field-effect transistors (TFETs) are a better choice than those based on MOSFETs. This was attributed to the smaller response time and lower leakage of TFET biosensors. In recent years, various types of TFET-based biosensors with different architectures such as core–shell nanotubes^5^, vertical^6–8^ and bilayer^9^ structures, have been proposed. In 2008, Hueting et al. proposed the first charge plasma-based diode in which metals with appropriate work functions induced the electrons and holes in an intrinsic semiconductor instead of using dopants^10^. The mentioned technique can be a viable solution for dopant-related problems in nanoscale transistors^11^. In 2013, Kumar and Janardhanan suggested the first silicon-based doping-less TFET, which paved the path for developing this idea^12^. Sharma et al. have proposed a doping-less TFET with drain current sensitivity of about 3 × 10^4^ at *V*_*GS*_ = 1.2 V^13^. Mahalaxmi et al. have developed a dual-metal-gate doping-less TFET and the drain current sensitivity of about 5 × 10^8^ was achieved^14^. In 2022, we proposed the first doping-less biosensor based on the cladding layer concept in which a highly-doped semiconductor acts as an inductive metal in the source region, and the drain current sensitivity of 6.17 × 10^5^ at *V*_*GS*_ = 0.4 V was obtained^15^. While trap-assisted tunneling is expected to cause lower problems in the doping-less TFET compared with the doping-based ones, their negative effects on the performance of biosensors should not be neglected. In this paper, we propose a novel biosensor which benefits from a doping-less tunneling junction that is built over an n^+^-drain region. Our paper's first aim is to assess our biosensor’s performance and investigate its reliability in the presence of TAT**.**

## Device structure and simulation methodology

**Figure 1:**
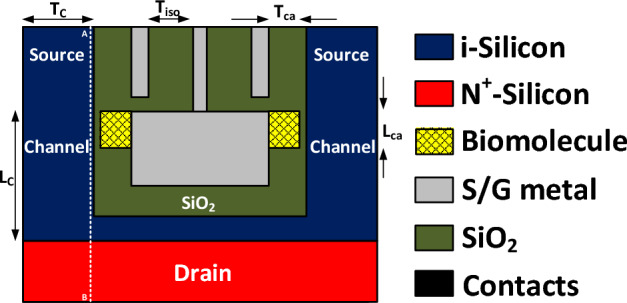
A schematic cross-sectional view of the proposed DMDS-TFET biosensor structure.

In Fig. 1, a cross-sectional view of our Dielectric-Modulated Dual doping-less Source TFET-based (DMDS-TFET) biosensor is depicted. In our device, which benefits from two source regions, carriers tunnel to a U-shape channel and then move toward the drain side, which is located at the bottom of the biosensor. To convert this TFET to a biosensor, two cavities with the dimension of 5 nm × 20 nm are carved in the gate dielectric using the wet-etching technique^16^. The channel length and thickness are 50 nm and 10 nm, respectively. The work function of the gate metal is 4.3 eV, while Platinum, with the work function of 5.93 eV, induces holes in the source region. Although the tunneling interface of our biosensor is intrinsic, the drain region is n^+^-doped with a concentration of 3 × 10^18^. Due to using silicon and SiO_2_ in the design of this biosensor, its fabrication process is fully compatible with CMOS technology. To prevent gate-leakage current 1 nm distance between the cavities and the channel is devised^17^. We also use a 20 nm distance (T_iso_) between the gate and source metals.

**Figure 2:**
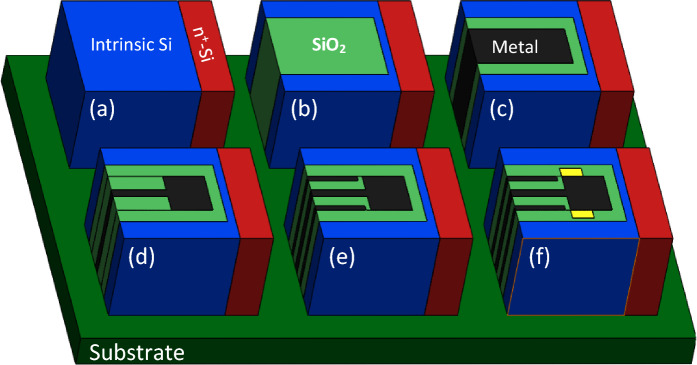
Fabrication process steps for realizing DMDS-TFET structure.

Although the structure of the proposed biosensor geometrically seems a little complex, but from the employed materials point of view, it is compatible with conventional CMOS technology. According to Fig. 2 we propose a multi-step fabrication process to realize DMDS-TFET. It commences with the epitaxial growth of n^+^ silicon next to an intrinsic silicon layer (see Fig. 2a). The selective etch technique is employed to create a U-shaped trench in the intrinsic silicon, followed by the deposition of SiO_2_ in the U-shaped trench, which acts as the gate spacer (see Fig. 2b). In the next step, another trench is created in the gate spacer, which is filled with the gate metal using the deposition technique (see Fig. 2c). Then, two formed trenches are filled with SiO_2_ (see Fig. 2d). The other trenches are created in the spacer regions for the deposition of source metals (see Fig. 2e). Finally, the wet etching technique is employed to shape two cavities in the channel (see Fig. 2f).

**Figure 3:**
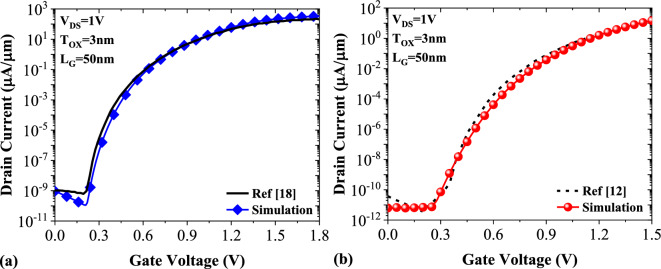
Reproduction of the transfer characteristics of (**a**) a double-gate TFET^18^ and (**b**) a doping-less TFET^12^ by our calibrated simulation framework.

In Fig. 3a, we have drawn the extracted values of the transfer characteristics of ref^18^ alongside our reproduced results, and a good matching is obtained for all bias points. Since our device simulator does not have an appropriate carrier-induced bandgap narrowing model, we chose another TFET based on the charge plasma concept^12^ for calibration, too (see Fig. 3b). A reasonable match between the original and the regenerated sets of data indicates that our following reported performance evaluations are reasonably valid and reliable.

Silvaco ATLAS device simulator was employed for the simulation of our proposed biosensor^19^. Due to the higher accuracy of the dynamic non-local BTBT model compared with local models, we used this model for calculating the on-state current. We have also activated auger, SRH, CVT, fermi, and drift–diffusion models for all the simulations. Due to the channel thickness of 10 nm and a single gate architecture, subband quantization was neglected in the simulations. Since m_e_ = 0.22m_0_ and m_h_ = 0.17m_0_ were used for the calibration stage, we have used the mentioned values to simulate DMDS-TFET biosensor. Our main aim in this paper is to thoroughly evaluate the impact of trap-assisted tunneling (TAT), one of the tunneling transistors' main drawbacks, on the TFET-based biosensors' performance^20^. So, we calculate the impact of TAT on the FOMs, such as drain current sensitivity (${S}_{{I}_{ds}}$) and subthreshold swing sensitivity (${S}_{SS}$).

## Simulation results

**Figure 4:**
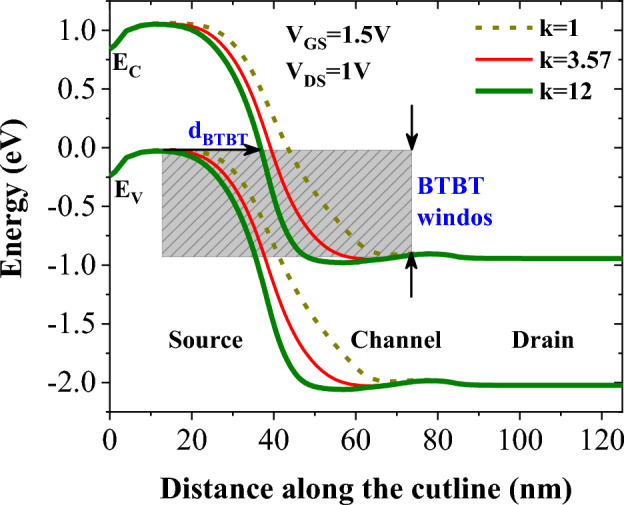
Impact of different biomolecules filling the cavities on the energy bands diagram.

The impact of various biomolecules on the energy bands diagrams of DMDS-TFET biosensor along the A-B cutline (drawn on Fig. 1) are illustrated in Fig. 4. According to this figure, when air is replaced with 3-aminopropyltriethoxysilane (APTES with *k* = 3.57) or Gelatin (with *k* = 12) biomolecules in the cavities the band-to-band tunneling distance (*d*_*BTBT*_) reduces. Such a reduction is mainly attributed to the impact of permittivity of the biomolecules on the electric field strength at the tunneling junction. When the cavities are filled with Gelatin, we have the lowest *d*_*BTBT*_, which means that the intensity of the electric field at the source-channel junction is significantly higher.

The equation that shows the dependence of tunneling probability on different parameters of the device is given by^18^

$$T(E) \propto exp\left( { - \frac{{4\sqrt {2m^{*} } E_{g}^{{3/2}} }}{{3\left| e \right|\hbar \left( {E_{g} + \Delta \Phi } \right)}}\sqrt {\frac{{\varepsilon _{c} }}{{\varepsilon _{{ox}} }}} t_{{ox}} t_{c} } \right)\Delta \Phi$$

where m* is the effective mass of charge carrier, *E*_*g*_ is bandgap, $\hslash$ is the reduced plank constant, *ΔΦ* is the energy overlap of the tunneling window, *ε*_*C*_ and *t*_*C*_ are the channel dielectric constant and thickness, *t*_*ox*_ is the dielectric thickness (in this work comprised of SiO_2_ and cavity thicknesses), and *ε*_*ox*_ is the dielectric constant. The exponential dependence of the tunneling probability on the dielectric constant of biomolecules indicates that biomolecules with higher dielectric constants remarkably enhance tunneling probability, resulting in higher on-state current.

**Figure 5:**
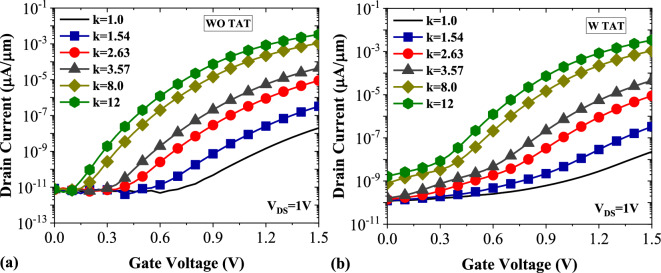
Transfer characteristics of DMDS-TFET (**a**) without TAT (**b**) with TAT.

Figure 5 compares the transfer characteristics of DMDS-TFET biosensor at the presence of different biomolecules with and without undesirable TAT conduction mechanism. Comparing these two figures shows that including TAT model in the simulations increases the biosensor's off-state current. Furthermore, TAT significantly modulates the onset voltage of tunneling. While in Fig. 5a, there is a distinct boundary between the off-state and on-state even for *k* = 1, in Fig. 5b, the gradual increase of the drain current makes it difficult to clearly distinguish these states except for *k* = 8 and *k* = 12. This means that excluding TAT from the simulations can lead to more ideal but unrealistic results.

**Figure 6:**
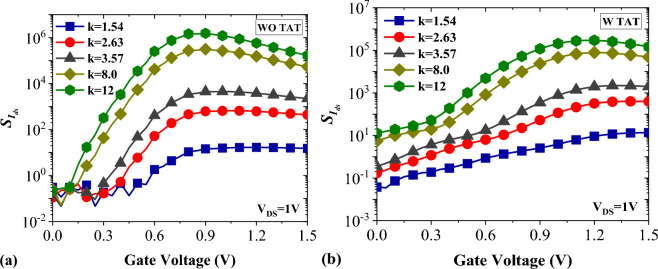
Drain current sensitivity of DMDS-TFET (**a**) without TAT (**b**) with TAT.

Drain current sensitivity is one of the main merit factors in the performance assessment of FET-based sensor. It is given by

$${S}_{{I}_{ds}}=\left(\frac{{I}_{ds}^{bio}-{I}_{ds}^{air}}{{I}_{ds}^{air}}\right)$$

where ${I}_{ds}^{air}$ is the drain current of the device with air-filled cavities and ${I}_{ds}^{bio}$ is the drain current in the presence of biomolecules with *k* values higher than the air^21^. Figure 6a,b illustrates the drain current sensitivity for various biomolecules with and without TAT. It can be seen that including TAT in the simulations reduce the ${S}_{{I}_{ds}}$ of the biosensor. Moreover, TAT Shifts the ${S}_{{I}_{ds.max}}$ point to the higher gate voltages. According to the left figure, for *k* = 12, we have ${S}_{{I}_{ds.max}}$ = 1.53 × 10^6^ at *V*_*GS*_ = 0.9 V, while on the other hand and for the same *k*, TAT degrades the ${S}_{{I}_{ds.max}}$ to 2.94 × 10^5^ at *V*_*GS*_ = 1.2 V.

**Figure 7:**
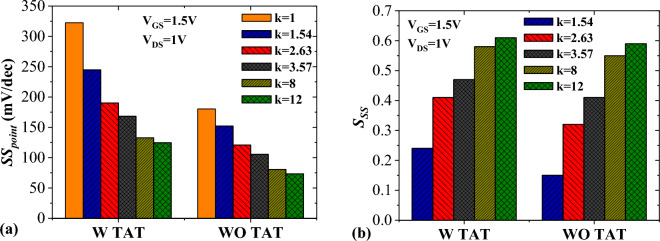
Impact of TAT on (**a**) subthreshold swing and (**b**) subthreshold swing sensitivity.

The negative impact of trap states that facilitate undesired tunneling of charge carriers from the source valance band to the channel conduction band is undeniable. One the manifestations of this phenomenon is the change in the steepness of the device switching. In order to study the switching behavior of the TFET sensor, we compare the subthreshold swing of the device, with and without TAT, in Fig. 7a. It can be inferred that including TAT dramatically decreases the subthreshold swing for all values of *k*. For example, there is 142.1 mV/dec difference between the values of subthreshold swing without and with TAT for *k* = 1. While this difference reaches 51.29 mV/dec for *k* = 12. Subthreshold swing sensitivity is defined by

$${S}_{SS}=\left|\frac{{SS}_{air}-{SS}_{bio}}{{SS}_{air}}\right|.$$

where *SS*_*air*_ and *SS*_*bio*_ are the subthreshold swing of the device with air-filled and biomolecule-filled cavities, respectively^21^. One interesting point is that, unlike the ${S}_{{I}_{ds}}$, in this case, the values of ${S}_{SS}$ in the presence of TAT are higher than the values of ${S}_{SS}$ when TAT is neglected (see Fig. 7b). This is mainly originated from the wide differences among the subthreshold swings when the TAT model is activated.

The selectivity of our biosensor is also investigated by calculating the selectivity factor between APTES-Biotin, and Biotin-Uricase, respectively, according to the following equations^22^,

$${\Delta S}_{1}=\frac{{I}_{ds.APTES}-{I}_{ds.Biotin}}{{I}_{ds.Biotin}}$$

$${\Delta S}_{2}=\frac{{I}_{ds.Biotin}-{I}_{ds.Uricase}}{{I}_{ds.Uricase}}.$$

**Figure 8:**
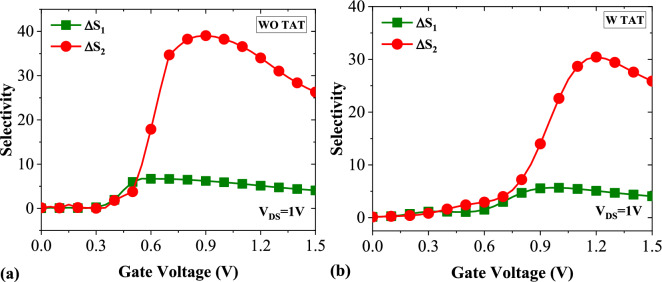
The selectivity between [APTES-Biotin] and [Biotin-Uricase] (**a**) without TAT (**b**) with TAT.

Figure 8a illustrates the selectivity between APTES and Biotin (${\Delta S}_{1}$) and the selectivity between Biotin and Uricase (${\Delta S}_{2}$) in the absence of the TAT mechanism, while Fig. 8b portrays these parameters in the presence of TAT. Both figures depict that our biosensor is more capable of distinguishing between Biotin and Uricase than APTES and Biotin. This can be attributed to the relative difference between the dielectric constant of two biomolecules which is 0.7 for the former and 0.35 for the latter.

**Figure 9:**
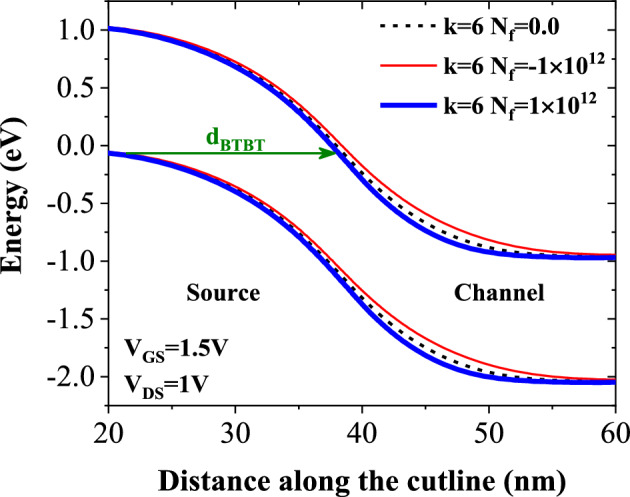
Impact of charge biomolecule of DNA on the energy bands diagram.

**Figure 10:**
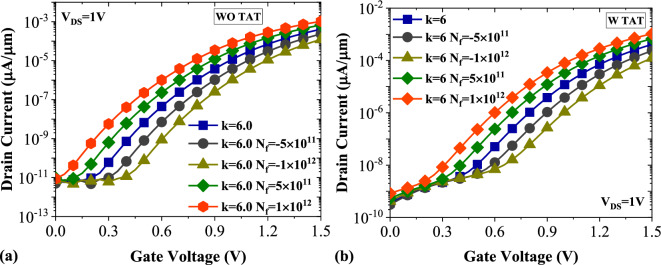
Transfer characteristics of DMDS-TFET biosensor (**a**) without TAT and (**b**) with TAT for charged DNA biomolecule.

One distinguishing feature of FET-based biosensors is their ability to detect charged biomolecules in addition to neutral biomolecules. In this section, we evaluate our biosensor's performance in detecting DNA biomolecules (with *k* = 6). Figure 9 shows the energy bands diagram at the tunneling junction along the A-B cutline (as depicted in Fig. 1). The figure indicates that positively charged biomolecule forms a steeper tunneling junction which can decrease band-to-band tunneling distance at the source-channel junction, while negatively charged biomolecule degrades band bending at the tunneling junction, leading to a higher *d*_*BTBT*_. Figure 10a,b compares the transfer characteristics of positively and negatively charged DNA biomolecules without and with TAT. In Fig. 10a, we have a much-steeper switching and lower values of *V*_*onset*_ (the gate voltage at which BTBT starts). While by taking TAT into account *V*_*onset*_ increases considerably. For example, there is a *ΔV*_*onset*_ of 0.23 V for *k* = 6 and *N*_*f*_ = 1 × 10^12^ (C cm^−2^) between the two cases.

**Figure 11:**
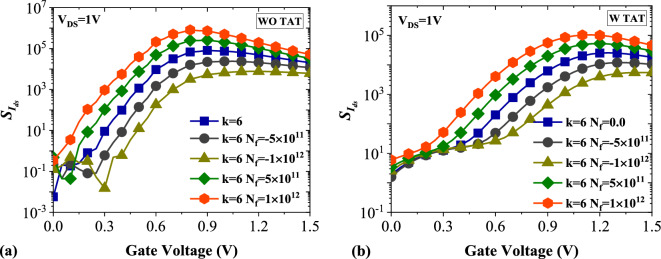
Drain current sensitivity of DMDS-TFET biosensor (**a**) without TAT and (**b**) with TAT for charged DNA biomolecule.

In Fig. 11a,b, the drain current sensitivity (${S}_{{I}_{ds}}$) of DMDS-TFET for charged DNA biomolecule without and with TAT is demonstrated. In the case of positively charged DNA with *N*_*f*_ = 1 × 10^12^ (C cm^−2^) and without TAT, the ${S}_{{I}_{ds.max}}$ can be as much as ${S}_{{I}_{ds.max}}$ for *k* = 12. In contrast, this value for negatively charged DNA with *N*_*f*_ = − 1 × 10^12^ (C cm^−2^) is almost the same as that for *k* = 3.57. As depicted in Fig. 11b, activating TAT reduces the ${S}_{{I}_{ds}}$ considerably. Interestingly, similar to the previous case, with including TAT in the simulations, the values of ${S}_{{I}_{ds.max}}$ for positively charged DNA with *N*_*f*_ = 1 × 10^12^ (C cm^−2^) are close to the ${S}_{{I}_{D}}$ for *k* = 8. In comparison, the values of ${S}_{{I}_{ds.max}}$ for negatively charged DNA with *N*_*f*_ = − 1 × 10^12^ (C cm^−2^) are marginally similar to that for *k* = 3.57.

**Figure 12:**
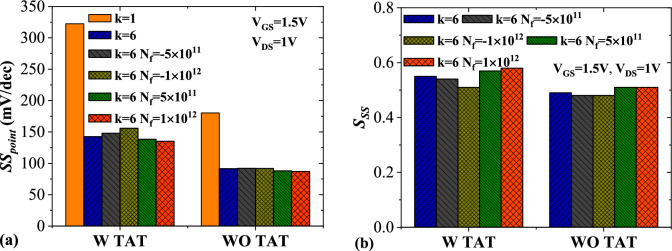
Impact of TAT on (**a**) subthreshold swing and (**b**) subthreshold swing sensitivity of the biosensor at the presence of charged DNA biomolecule.

Figure 12a shows the impact of trap-assisted tunneling on the subthreshold swing of DMDS-TFET at the presence of positively and negatively charged DNA biomolecules. Similar to the neutral biomolecules, activating TAT in the simulation degrades subthreshold swing considerably. It can be observed that the *SS* value for* k* = 6 and *N*_*f*_ = 1 × 10^12^ (C cm^−2^) is 87.2 mV/dec, and with activating TAT, the value with 154% increase reaches 135.1 mV/dec. In Fig. 12b, the subthreshold swing sensitivity (${S}_{SS}$) of the biosensor for charged DNA with and without TAT activation is plotted. Like the neutral biomolecules, the higher values of ${S}_{SS}$ in the presence of TAT are mainly attributed to the wide differences among the subthreshold swings when the TAT model is activated. For *k* = 6 and *N*_*f*_ = 1 × 10^12^ (C cm^−2^), The ${S}_{SS}$ = 0.58 and excluding TAT decreases the value to ${S}_{SS}$ = 0.51.

**Figure 13:**
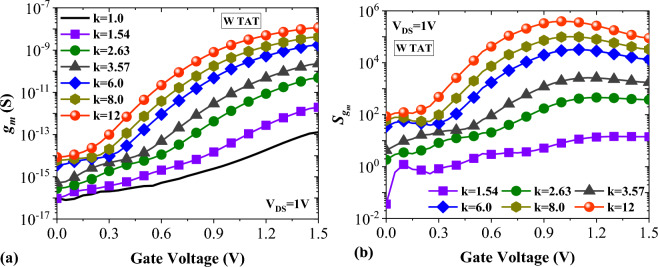
Impact of TAT on (**a**) transconductance and (**b**) transconductance sensitivity for various biomolecules.

A practical biosensor should have high linearity and small distortion. Reduction of device linearity can lead to the degradation of signal-to-noise performance, which decrease biosensors sensitivity. Calculating transconductance is one way to assess these parameters^23^. Figure 13a shows the impact of TAT on the transconductance of DMDS-TFET biosensor. It can be seen that higher values of *k* contribute to higher transconductance. To attain more realistic results, TAT is also activated for this graph. In Fig. 13b, the transconductance sensitivity for different values of *k* is plotted. It is formulated as

$${S}_{{g}_{m}}=\left|\frac{{{S}_{g}}_{m.air}-{{S}_{g}}_{m.bio}}{{{S}_{g}}_{m.air}}\right|$$

where ${{S}_{g}}_{m.air}$ is the value of the transconductance for *k* = 1 and ${{S}_{g}}_{m.bio}$ is the value of the transconductance at the presence of biomolecule.

**Figure 14:**
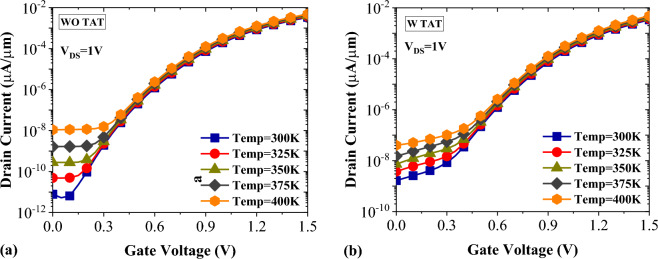
Impact of temperature on the transfer characteristics of DMDS-TFET biosensor (**a**) without TAT and (**b**) with TAT.

In Fig. 14 we investigate the sensor stability in the presence of temperature change. Temperature change is an important non-ideality that can considerably degrade the TFET-based biosensors performance stability. The impact of 100 K increment in the temperature for Gelatin (with k = 12), which has the highest off-state current compared to the other biomolecules, is evaluated in the figure. It can clearly be seen that in both figures subthreshold region is more affected. This is mainly because the band-to-band tunneling equation has no direct dependency on temperature.

In Table 1 we compare the performance of some recently reported dielectric-modulated biosensors. For this purpose, the Gelatin biomolecule (with k = 12) is chosen, and the biosensors' threshold voltage sensitivity and off-state current sensitivity are compared. The table indicates that the DMDS-TFET biosensor can be considered as one of the best proposed biosensors ever.

**Table 1:** Performance comparison of some dielectric-modulated biosensor for Gelatin biomolecule.

Refs./Year	Architecture	Material	${S}_{{I}_{off}}$	${S}_{{V}_{th}}$	*V*_*Bias*_ (V)
^24^/2019	Double gate junction-less TFET	Si	~ 100	28.57	1.2
^25^/2021	Extended gate HTFET	InGaAs/Si	90	–	1.5
^26^/2021	FinFET	GaAs_1−x_Sb_x_	98.4	26.34	1
^27^/2021	Negative capacitance FinFET	Si	99.99	295.89	1
This Work	Vertical dual Doping-less tunneling junction	Si	98.58	50.27	1.5

**Figure 15:**
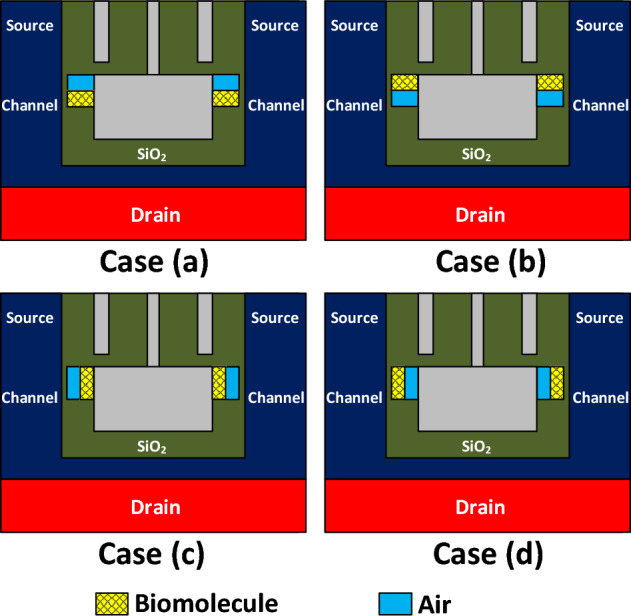
Various types of semi-filled cavities.

**Figure 16:**
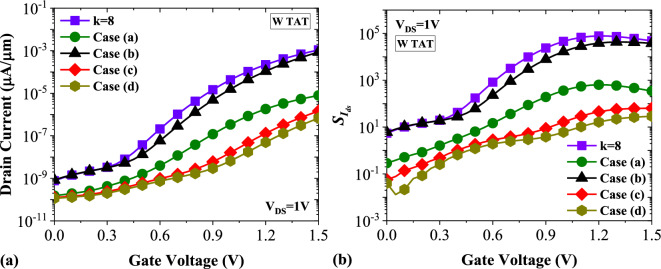
Impact of various cases of semi-filled cavities on (**a**) transfer characteristics and (**b**) drain current sensitivity of DMDS-TFET biosensor.

Although, from the beginning of the article to this point, we have considered fully filled cavities with the filling factor of 100% in all simulations, the existence of unfilled regions in the cavities may degrade the biosensor's performance^28^. To elucidate the impact of semi-filled cavities, we address four different configurations, plotted in Fig. 15. In Fig. 16a, the impact of Keratin biomolecule with a filling factor of 50% on the transfer characteristics of DMDS-TFET is depicted. It is evident that in case (b), the on-state current of the biosensor is close to the case in which the filling factor is 100%. While in cases (c) and (d), the drain current decreased significantly. This is mainly because, in these mentioned cases, the capacitive coupling of the gate with the tunneling junction has dropped. Figure 16b shows the impact of four semi-filled cases with the filling factor of 50% on the drain current sensitivity. In this figure, we have a ${S}_{{I}_{ds}}$ = 3.78 × 10^4^ for case (b), which is marginally close to the case with FF = 100%. At the same time, these values for cases (**c**) and (**d**) reach 63.54 and 28.91, respectively.

## Conclusion

A novel TFET-based biosensor that benefits from dual doping-less tunneling junction is suggested. In this device, a U-shape channel connects source regions to an n^+^-doped drain region. Due to using silicon and SiO_2_ in our biosensor, it is fully compatible with CMOS technology. Various neutral biomolecules, such as Uricase and Biotin, and charged DNA biomolecule were separately inserted into the cavities and the performance of the biosensor was evaluated by simulations. All the simulations were done by Silvaco ATLAS device simulator which had been calibrated by the valid data of the similar structure. We have shown that the role of trap-assisted tunneling, even in a doping-less tunneling junction, cannot be neglected. The impact of TAT on parameters like *I*_*on*_ and subthreshold swing was calculated, and unignorably discrepancies compared with the cases in which TAT was not included have been observed. The realistic FOMs such as ${S}_{{I}_{ds}}$ = 1.48 × 10^5^, and ${S}_{SS}$ = 0.61 illustrate that the performance of our biosensor is acceptable for high-sensitivity applications.

## Data Availability

The datasets used and/or analyzed during the current study available from the corresponding author on reasonable request.

## References

[1] Jang D-Y, Kim Y-P, Kim H-S, Ko Park S-H, Choi S-Y, Choi Y-K (2007). Sublithographic vertical gold nanogap for label-free electrical detection of protein-ligand binding. J. Vac. Sci. Technol. B.

[2] Stern E, Klemic JF, Routenberg DA, Wyrembak PN, Turner-Evans DB, Hamilton AD, LaVan DA, Fahmy TM, Reed MA (2007). Label-free immunodetection with CMOS-compatible semiconducting nanowires. Nature.

[3] Im H, Huang X-J, Gu B, Choi Y-K (2007). A dielectric-modulated field-effect transistor for biosensing. Nat. Nanotechnol..

[4] Gedam A, Acharya B, Mishra GP (2021). Design and performance assessment of dielectrically modulated nanotube TFET biosensor. IEEE Sens. J..

[5] Shreya S, Khan AH, Kumar N, Amin SI, Anand S (2019). Core-shell junctionless nanotube tunnel field effect transistor: Design and sensitivity analysis for biosensing application. IEEE Sens. J..

[6] Wang Y, Li C, Li O, Cheng S, Liu W, You H (2022). Simulation study of dual metal-gate inverted T-shaped TFET for label-free biosensing. IEEE Sens. J..

[7] Cherik IC, Mohammadi S (2022). Vertical tunneling field-effect transistor with germanium source and T-shaped silicon channel for switching and biosensing applications: A simulation study. IEEE Trans. Electron. Devices.

[8] Priyadarshani KN, Singh S (2021). Ultra sensitive label-free detection of biomolecules using vertically extended drain double gate SiO_5_GeO_5_ source tunnel FET. IEEE Trans. NanoBiosci..

[9] Palepu J, Patel S, Sinha S, Mallidi RK, Karthik GVN, Majumdar B, Mukhopadhyay S, Kanungo S (2023). Investigation of the dielectrically modulated electron hole bilayer tunnel field effect transistor for biomolecule detections. Curr. Appl. Phys..

[10] Hueting RJ, Rajasekharan B, Salm C, Schmitz J (2008). The charge plasma PN diode. IEEE Electron. Device Lett..

[11] Chiang M-H, Lin J-N, Kim K, Chuang C-T (2007). Random dopant fluctuation in limited-width FinFET technologies. IEEE Trans. Electron. Devices.

[12] Kumar MJ, Janardhanan S (2013). Doping-less tunnel field effect transistor: Design and investigation. IEEE Trans. Electron. Devices.

[13] Sharma D, Singh D, Pandey S, Yadav S, Kondekar P (2017). Comparative analysis of full-gate and short-gate dielectric modulated electrically doped Tunnel-FET based biosensors. Superlattices Microstruct..

[14] Acharya B, Mishra GP (2020). Design and analysis of dual-metal-gate double-cavity charge-plasma-TFET as a label free biosensor. IEEE Sens. J..

[15] Cherik IC, Mohammadi S (2022). Dielectric modulated doping-less tunnel field-effect transistor, a novel biosensor based on cladding layer concept. IEEE Sens. J..

[16] Chandan BV, Nigam K, Sharma D (2018). Junctionless based dielectric modulated electrically doped tunnel FET based biosensor for label-free detection. Micro Nano Lett..

[17] Kanungo S, Chattopadhyay S, Gupta PS, Sinha K, Rahaman H (2016). Study and analysis of the effects of SiGe source and pocket-doped channel on sensing performance of dielectrically modulated tunnel FET-based biosensors. IEEE Trans. Electron. Devices.

[18] Boucart K, Ionescu AM (2007). Double-gate tunnel FET with high-$\kappa $ gate dielectric. IEEE Trans. Electron. Devices.

[19] Silvaco, ATLAS Device Simulation Software User’s Manual, no. version 3.2. (2015).

[20] Sant, S., Schenk, A., Moselund, K. & Riel, H. Impact of trap-assisted tunneling and channel quantization on InAs/Si hetero tunnel FETs. In *2016 74th Annual Device Research Conference (DRC)* 1–2 (IEEE). 10.1109/DRC.2016.7548413 (2016).

[21] Patil M, Gedam A, Mishra GP (2020). Performance assessment of a cavity on source ChargePlasmaTFET-based biosensor. IEEE Sens. J..

[22] Dwivedi P, Singh R (2020). Investigation the impact of the gate work-function and biases on the sensing metrics of TFET based biosensors. Eng. Res. Express.

[23] Verma M, Tirkey S, Yadav S, Sharma D, Yadav DS (2017). Performance assessment of a novel vertical dielectrically modulated TFET-based biosensor. IEEE Trans Electron Devices.

[24] Wadhwa G, Raj B (2019). Design, simulation and performance analysis of JLTFET biosensor for high sensitivity. IEEE Trans. Nanotechnol..

[25] Mukhopadhyay S, Sen D, Goswami B, Sarkar SK (2020). Performance evaluation of dielectrically modulated extended gate single cavity InGaAs/Si HTFET based label-free biosensor considering non-ideal issues. IEEE Sens. J..

[26] Dixit A, Samajdar DP, Bagga N (2021). Dielectric modulated GaAs1−x Sb X FinFET as a label-free biosensor: Device proposal and investigation. Semicond. Sci. Technol..

[27] Dixit A, Samajdar DP, Chauhan V (2021). Sensitivity analysis of a novel negative capacitance FinFET for label-free biosensing. IEEE Trans Electron Devices.

[28] Narang R, Saxena M, Gupta M (2015). Comparative analysis of dielectric-modulated FET and TFET-based biosensor. IEEE Trans. Nanotechnol..

